# Exploring patient, caregiver and care team experiences of destination therapy Left Ventricular Assist Device (LVAD): A multiple case qualitative study protocol

**DOI:** 10.1016/j.ijnsa.2026.100572

**Published:** 2026-05-23

**Authors:** Nathalie Rebetez, Maya Zumstein-Shaha, Tania Dominguez Canosa, Yohanna Guyon, Claudia Ortoleva Bucher

**Affiliations:** a School of Health Sciences Fribourg, HES-SO, University of Applied Sciences and Arts Western Switzerland, Fribourg, Switzerland; b Bern University of Applied Sciences, Department of Health Professions, Bern, Switzerland; c Institute of Higher Education and Research in Healthcare (IUFRS), University of Lausanne, Lausanne, Switzerland; d Department Surgery and Cardiology, Lausanne University Hospital, Lausanne, Switzerland

**Keywords:** Ventricular assist devices, Heart failure, Nursing care, Caregivers, Family relations, Patient education as topic, Qualitative research

## Abstract

**Background:**

Advanced heart failure constitutes a major public health issue. For patients ineligible for heart transplantation, the Left Ventricular Assist Device (LVAD) as Destination Therapy (DT) is a therapeutic option that prolongs survival, at the cost of significant constraints and risks of complications. The lived experience of patients, caregivers and the interprofessional team remains insufficiently documented, particularly in the Swiss context.

**Objective:**

To explore the lived experience, needs and coping strategies of LVAD-DT patients, their family caregivers, dyadic/triadic functioning, and the perceptions of healthcare professionals involved in their care.

**Methods and analysis:**

This exploratory qualitative study uses a multiple case study design grounded in a social constructivist paradigm. Data will include individual and dyadic/triadic semi-structured interviews, one focus group with healthcare professionals, sociodemographic and clinical data, as well as field notes. Qualitative data will be treated with thematic analysis according to Braun and Clarke (2006), combining inductive and deductive approaches, and into both intra- and inter-case analyses.

**What is already known**

• LVAD destination therapy improves survival in advanced heart failure but entails major long-term psychosocial and self-management challenges.

• Family caregivers are central to daily LVAD care and experience substantial burden.

• Evidence informing nursing practice and dyadic/triadic care in LVAD-DT remains limited.

**What this paper adds**

• A qualitative dyadic/triadic multiple-case protocol including patients, family caregivers and healthcare professionals.

• A clear focus on nursing competencies, therapeutic education and care coordination in LVAD-DT.

• An explicit goal to inform the development of a future dyadic/triadic nursing intervention.

Alt-text: Unlabelled box dummy alt text

## Background

### Advanced heart failure and left ventricular assist device (LVAD)

Heart Failure is a major global health issue due to its high prevalence, morbidity, mortality and associated costs (Kępińska et al., 2019). Despite therapeutic advances, many patients progress to advanced heart failure that is refractory to pharmacological therapies (Regamey et al., 2018). In this context, heart transplantation and the implantation of a LVAD represent the main therapeutic options (Bouayed et al., 2024; Regamey et al., 2018). The LVAD may be implanted as a temporary solution (Bridge to Transplant – BTT) or as a definitive therapy (Destination Therapy – DT) for patients ineligible for transplantation (Bouayed et al., 2024; Regamey et al., 2018). Current data show a survival of around 73% at two years and 52% at five years among LVAD-DT patients, at the cost of a high risk of complications and psychosocial constraints (Finch et al., 2023; Mehra et al., 2019; Melendo-Viu et al., 2023; Wasilewski et al., 2024). The increasing use of LVAD-DT is partly due to persistent donor shortages and the ageing heart failure population, reinforcing the need to develop long-term adapted care models (Saeed et al., 2023).

### Lived experience and symptoms of LVAD-DT patients

Beyond survival, improving quality of life is a central objective (Inyom et al., 2022; Rapelli et al., 2023). Patients often assess their quality of life by comparing their situation before and after implantation (Krimminger and Sledge, 2022). Daily management of the device requires complex technical and organisational skills, generating stress, uncertainty and constant vigilance (Kitko et al., 2016; Standing et al., 2018). LVAD-DT is frequently perceived as a “second chance”, while imposing a physical, psychosocial and existential burden (Inyom et al., 2022; Rapelli et al., 2023). This ambivalence reflects a complex chronic disease trajectory requiring continuous adjustment (Levelink and Brütt, 2021; Standing et al., 2018).

### Role of the family caregiver and the interprofessional team

LVAD-DT patients depend heavily on their family caregivers, whose role is central in device management, symptom monitoring and decision-making (Krimminger and Sledge, 2022; Lyons et al., 2023). This interdependence is recognised in clinical guidelines, which consider the presence of a reliable family caregiver as an essential criterion for LVAD implantation (Saeed et al., 2023). The dyad/triad comprising the LVAD-DT patient and their family caregiver(s) relies on close coordination, effective communication and shared decisions (Rapelli et al., 2023).

However, this responsibility exposes family caregivers to significant emotional, physical and logistical burden, affecting quality of life (Inyom et al., 2022; Krimminger and Sledge, 2022; Lyons et al., 2023). Interventions focusing exclusively on the patient show limited effects, partly due to insufficient consideration of dyadic/triadic functioning. The lack of structured family caregiver support is also associated with increased risk of complications, hospitalisations and self-management failures, reinforcing the need to consider family caregivers as full intervention targets (Adams and Wrightson, 2018; Buck et al., 2018; Kitko et al., 2016; Saeed et al., 2023).

Support for dyads/triads is provided by an interprofessional team. A joint understanding of the lived experience of LVAD-DT patients, family caregivers and healthcare professionals is therefore essential to adapt educational and preventive interventions in the LVAD-DT context (Buck et al., 2018; Streur et al., 2020). LVAD-DT care relies heavily on advanced nursing competencies, long-term therapeutic education and coordination across care settings.

### Gap

Current interventions, focused on education and behavioural recommendations, remain limited (Bidwell et al., 2017; D’Andria Ursoleo et al., 2024). LVAD-DT patients and their family caregiver(s) express the need for holistic care adapted to their physical, emotional and psychosocial needs (Bouayed et al., 2024; Kitko et al., 2016; Rapelli et al., 2023). Despite increasing use of LVAD-DT, the literature on the shared experience of LVAD-DT patients, their family caregivers and healthcare professionals remains limited. To date, no study has explored this experience in the Swiss context. An in-depth and contextualised understanding is needed to support the development of appropriate interventions for the complex care situations associated with LVAD-DT. This study is explicitly positioned to inform nursing practice, education and the development of complex nursing interventions in advanced heart failure and mechanical circulatory support.

### Aim

This study aims to explore the lived experience, needs and coping strategies of LVAD-DT patients, their family caregiver(s) and the dyad/triad, as well as the perceptions of healthcare professionals involved in their care. To generate empirical knowledge to inform the future development of a dyadic/triadic nursing intervention supporting LVAD-DT care.

## Methods

### Study design and case definition

This is an exploratory qualitative study using a multiple-case design grounded in the social constructivist paradigm (Talbot, 2017). Cases are defined as dyads/triads composed of an LVAD-DT patient and their family caregiver(s). Between 6 and 8 units of analysis will be studied, consistent with Yin’s analytic replication logic (Yin RK, 2018).

### Context & study timeline

The study will take place at Lausanne University Hospital (CHUV), within the cardiology outpatient clinic, starting from the first post-implantation ambulatory consultation. The project is planned over two years. Data collection using a purposive sample will take place during summer until end of 2026, using individual and dyadic/triadic semi-structured interviews and focus groups, which will be transcribed verbatim. Thematic analysis will be applied. Analysis and dissemination will continue through 2027–2028.

### Population and sampling

Four subgroups will be included: i. LVAD-DT patients ; ii. Family caregivers ; iii. Dyads/triads (LVAD-DT patient– family caregiver(s)) ; iiii. Healthcare professionals involved in their care (nurses, physicians, perfusionists). A convenience sampling approach will be used. Inclusion and exclusion criteria are defined for the study (Table 1).

**Table 1:** Inclusion and exclusion criteria.

Population	Inclusion Criteria	Exclusion Criteria
LVAD-DT patients	ο Age ≥18 ο First ambulatory visit completed ο Written consent ο Understands and speaks French ο Family caregiver participates in the study	ο Lack of discernment ο Does not speak French
Family caregivers	ο Designated by LVAD-DT patient ο Written consent ο Understands and speaks French	ο Lack of discernment ο Does not speak French
Healthcare professionals	ο Involved in LVAD-DT patient care for ≥2 months ο Direct involvement with dyad/triad ο Written consent ο Speaks French	ο Temporary workers or students

### Patient and public involvement

A patient partner living with an LVAD is integrated into the research team. This patient partner reviewed the study protocol and provided feedback, opinions, and suggestions, particularly regarding the development of the interview guides for patients and family caregivers. Results will be disseminated to participants and stakeholders in an accessible format.

### Recruitment

LVAD-DT patients will be identified by LVAD nursing team at CHUV and contacted by the research team after their agreement. Family caregiver(s) will be identified by LVAD-DT patients and also contacted by the research team. Once consent is signed, interviews will take place based on participants’ availability and preferences (in person, video or telephone), ensuring flexibility and comfort (Carroll et al., 2022).

Healthcare professionals will be recruited after a project presentation by the research team, then selected according to inclusion criteria. They will participate in a focus group after providing informed consent.

### Data collection

Data will be collected at any point in the patient pathway following implantation, regardless of the implantation date and include:

• Sociodemographic and clinical data of LVAD-DT patients and family caregiver(s).
• Sociodemographic data of healthcare professionals.
• Individual and dyadic/triadic semi-structured interviews (45–90 min).
• Focus group for healthcare professionals (60 min).
• Observation notes and researcher field logs.

**Fig. 1:**
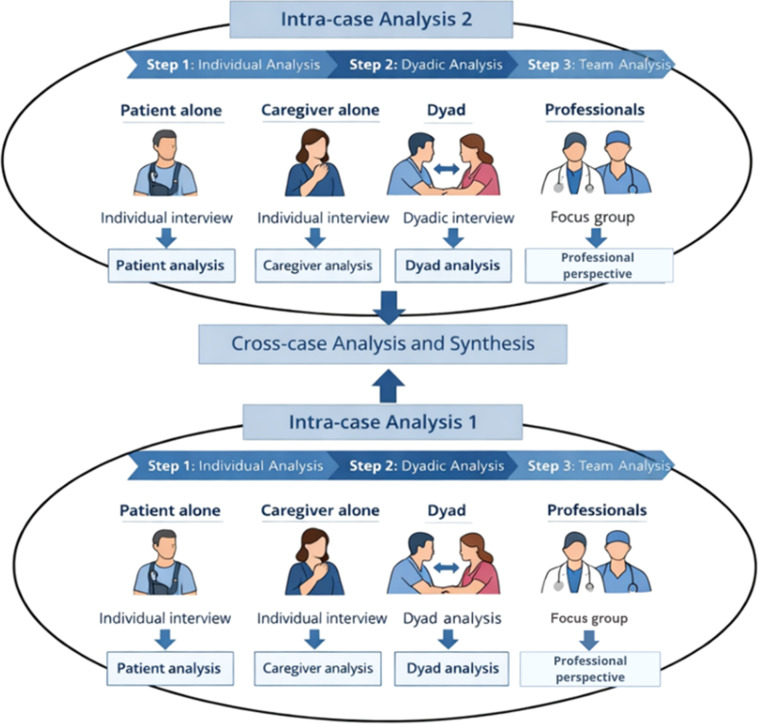
Diagram of the intra-case and inter-case analyses.

Interviews will be conducted separately to avoid cross-influences, and dyads/triads interviewed after individual interviews (Fig. 1). All interviews and the focus group will be audio-recorded. Triangulation of data sources aims to reinforce interpretative depth.

### Data analysis

Analysis will occur in two phases:

1. **The intra-case analysis:** will be based on the articulation of four complementary sources: the analysis of the LVAD-DT patient alone (individual interview), the analysis of each family caregiver alone (individual interview), the analysis of the dyad/triad (dyadic/triadic interview), and the analysis of the healthcare professionals perspective derived from the focus groups, all analysed according to the same methodological framework in order to identify convergences and divergences (Talbot, 2017; Yin RK, 2018). This procedure will be applied in an identical manner to each case.
2. **Inter-case analysis:** Intra-case results will be compared to identify similarities, differences and transversal patterns (Chowdhury and Shil, 2021).Quantitative data will be analysed using descriptive statistics. Qualitative data (verbatim, field notes, journals) will be thematically analysed according to Braun and Clarke (Braun and Clarke, 2006), combining inductive and deductive approaches, grounded in Neuman’s framework (Neuman, 2011). Two researchers will analyse data iteratively with a third reviewer to limit bias (Yin RK, 2018).

### Scientific rigor

A triangulation of data sources will be carried out, including the semi-structured interviews with LVAD-DT patients, family caregiver(s), and healthcare professionals, as well as observation notes and field journals, in order to cross perspectives and strengthen the validity of the results. The purposive sampling will ensure diversity of the studied cases, both in terms of sociodemographic profiles and time elapsed since LVAD-DT implantation, allowing coverage of a wide variety of experiences and contexts.

The analysis will follow both an intra-case approach, to examine each dyad/triad in depth as an interactive system, and an inter-case approach, to compare cases with one another and identify common themes as well as divergences. All interviews will be fully transcribed and anonymised in order to protect participant confidentiality and ensure data integrity. Finally, multiple researchers will be involved in the analysis process, enabling comparison of interpretations and limiting subjective bias (Talbot, 2017).

### Ethical considerations and data management

The protocol was approved by the Commission d’Éthique du canton de Vaud (Projet-ID 2026–00,386). All participants will provide free and informed consent after receiving written and oral information detailing the study objectives, the nature of the expected participation, and their rights. Participation is voluntary and participants may withdraw from the study at any time, without consequences and without impact on the care or services to which they are entitled. No financial compensation is planned.

The data will be anonymised by coding, stored on a secure institutional server, and accessible only to authorised members of the research team, who are bound by confidentiality obligations.

Data will be kept for a period of 10 years in accordance with current regulatory requirements, then destroyed.

Given the potential vulnerability of LVAD-DT patients and their family caregiver(s), special attention will be paid to the well-being of participants during data collection. Interviews may be interrupted or stopped at any time at the request of participants. A systematic debriefing will be conducted at the end of each interview to address participants’ wellbeing and reflect on the interview process. No experimental procedure or modification of the care pathway is induced by participation in the study.

Results will be presented in aggregated and contextualised form to ensure participant non-identifiability.

## Discussion

LVAD-DT constitute an increasingly used therapeutic option for people with advanced refractory heart failing, enabling improved survival and symptoms (Mehra et al., 2019; Saeed et al., 2023). However, living with an LVAD-DT implies major constraints and a persistent risk of complications, requiring close outpatient follow-up and strong long-term self-management capacity (Finch et al., 2023; Wasilewski et al., 2024).

These issues concern not only LVAD-DT patients but also family caregiver(s), whose role is central in daily device management. The literature highlights significant burden and psychological distress within dyads/triads, likely to influence clinical outcomes and quality of life (Kitko et al., 2016; Stahl et al., 2019). Despite these findings, the specific needs of dyads/triads living with LVAD-DT and their coping strategies remain poorly documented, particularly in the context of destination therapy and long-term follow-up (Koutsavli et al., 2023).

Existing interventions in LVAD care remain largely patient-centred, with limited consideration of dyadic or triadic functioning and insufficient integration of the interprofessional team, despite the high level of coordination required between patients, family caregivers and healthcare professionals ((Buck et al., 2018; Kitko et al., 2016). Integrating the perspective of healthcare professionals is essential to understand the clinical, educational and organisational.

This multiple-case qualitative study aims to deepen understanding of the lived experience, needs and coping strategies of LVAD-DT patients, their family caregiver(s) and the dyad/triad within a highly complex, technology-dependent chronic care context. By integrating patient, caregiver and healthcare professional perspectives, the study will generate nursing-relevant empirical knowledge to inform the future development of dyadic/triadic nursing interventions supporting long-term LVAD-DT care (Talbot, 2017; Yin RK, 2018). Such interventions align with current recommendations advocating integrated, person- and family-centred care in complex chronic diseases and may contribute to improving care experiences and long-term clinical outcomes (Buck et al., 2018).

## Declaration of generative AI and AI-assisted

The authors used ChatGPT and Copilot to improve the clarity and style of the text of the manuscript. After using this tool, the authors reviewed and edited the content as needed and take full responsibility for the content of the published article.

## Funding

This project was funded by an HES-SO grant (18-S25). The funder did not influence the results/outcomes of the study despite the author's affiliation with the funder.

## CRediT authorship contribution statement

**Nathalie Rebetez:** Writing – review & editing, Writing – original draft, Visualization, Validation, Supervision, Resources, Project administration, Methodology, Investigation, Formal analysis, Data curation, Conceptualization. **Maya Zumstein-Shaha:** Writing – review & editing, Supervision, Methodology, Conceptualization. **Tania Dominguez Canosa:** Writing – review & editing. **Yohanna Guyon:** Resources, Writing – review & editing. **Claudia Ortoleva Bucher:** Writing – review & editing, Validation, Supervision, Resources, Project administration, Methodology, Investigation, Funding acquisition, Formal analysis, Data curation, Conceptualization.

## Declaration of competing interest

The authors declare that they have no known competing financial interests or personal relationships that could have appeared to influence the work reported in this paper. This study was funded by HES-SO. The funder had no role in the study design, data collection, analysis, interpretation of data, or decision to submit the manuscript for publication.

## Appendix Supplementary materials

Image, application 1

## References

[bib0001] Adams E.E., Wrightson M.L. (2018). Quality of life with an LVAD : a misunderstood concept. Heart Lung..

[bib0002] Bidwell J.T., Lyons K.S., Mudd J.O., Gelow J.M., Chien C.V., Hiatt S.O., Grady K.L., Lee C.S. (2017). Quality of life, depression, and anxiety in ventricular Assist device therapy : longtidunal outcomes for patients and Family caregivers. J. Cardiovasc. Nurs..

[bib0003] Bouayed Y.H., Meyer D.P., Bendjelid P.K., Giraud D.R., Ellenberger D.C., Hagerman D.A., Reymond D.P. (2024). Prise en charge mécanique de l’insuffisance cardiaque avancée. Rev. Médic. Suiss..

[bib0004] Braun V., Clarke V. (2006). Using thematic analysis in psychology. Qual. Res. Psychol..

[bib0005] Buck H.G., Stromberg A., Chung M.L., Donovan K.A., Harkness K., Howard A.M., Kato N., Polo R., Evangelista L.S. (2018). A systematic review of heart failure dyadic self-care interventions focusing on intervention components, contexts, and outcomes. Int. J. Nurs. Stud..

[bib0006] Carroll A.J., Hahn E.A., Grady K.L. (2022). Research engagement and experiences of patients pre- and post-implant of a left ventricular assist device from the mechanical circulatory support measures of adjustment and quality of life (MCS A-QOL) study. Qual. Life Res..

[bib0007] Chowdhury A., Shil N.C. (2021). Thinking ‘qualitative’ Through a case study : homework for a researcher. Am. J. Qual. Res..

[bib0008] D’Andria Ursoleo J., Pieri M., Calvo F., Altizio S., Gramegna M., Pontillo D., Ajello S., Scandroglio A.M. (2024). Long-term quality of life, psychological distress, and caregiver burden in octogenarians with LVAD : a single-centre experience. Int. J. Artif. Organs.

[bib0009] Finch A.S., Mohseni M.M., Simon L.V., Finch J.G., Gordon-Hackshaw L.E., Klassen A.B., Mullan A.F., Barbara D.W., Sandefur B.J. (2023). Characteristics and outcomes of patients in the Emergency department with left Ventricular Assist devices. West J. Emerg. Med..

[bib0010] Inyom C., Haese T., Schoenrath F., Potapov E., Knierim J. (2022). Lived experiences of patients implanted with left ventricular assist devices. Heart Lung..

[bib0011] Kępińska K., Adamczak D.M., Kałużna-Oleksy M. (2019). Advanced heart failure—a review. Adv. Clin. Exp. Med..

[bib0012] Kitko L.A., Hupcey J.E., Birriel B., Alonso W. (2016). Patients’ decision making process and expectations of a left ventricular assist device pre and post implantation. Heart Lung..

[bib0013] Koutsavli D., Kalogianni A., Souvatzi E.B., Misouridou E., Toulia G., Papageorgiou D., Timmins F., Kelesi-Stavropoulou M., Kapadochos T., Charitos C., Stavropoulou A., Kaba E., Pavlatou N., Margari N., Parissopoulos S., ICU Follow-Up Care Lab, D. of N., University of West Attica, Athens (2023). The lived experience of patients with left ventricular assist devices : a systematic review of qualitative studies. Eur. Heart. J..

[bib0014] Krimminger D.M., Sledge J.A. (2022). A qualitative study of life with a left ventricular assist device as a bridge to transplant : a new normal. Intens. Crit. Care Nurs..

[bib0015] Levelink M., Brütt A.L. (2021). Factors influencing health-related quality of life of patients with a left ventricular assist device : a systematic review and thematic synthesis. Eur. J. Cardiovasc. Nurs..

[bib0016] Lyons K.S., Whitlatch C.J., Vest A.R., Upshaw J.N., Johnson S.H., Morelock J., Lee C.S. (2023). Taking care of us© (TCU) study protocol : feasibility and acceptability of a dyadic intervention for couples living with heart failure. Pilot. Feasib.. Stud..

[bib0017] Mehra M.R., Uriel N., Naka Y., Cleveland J.C., Yuzefpolskaya M., Salerno C.T., Walsh M.N., Milano C.A., Patel C.B., Hutchins S.W., Ransom J., Ewald G.A., Itoh A., Raval N.Y., Silvestry S.C., Cogswell R., John R., Bhimaraj A., Bruckner B.A., MOMENTUM 3 Investigators (2019). A fully magnetically levitated left Ventricular assist device—final report. N. Engl. J. Med..

[bib0018] Melendo-Viu M., Dobarro D., Raposeiras Roubin S., Llamas Pernas C., Moliz Cordón C., Vazquez Lamas M., Piñón Esteban M., Varela Martínez M.Á., Abu Assi E., Pita Romero R., Legarra Calderón J.J., Íñiguez Romo A. (2023). Left Ventricular assist device as a destination therapy : current situation and the importance of patient selection. Life (Basel).

[bib0019] Neuman B. (2011).

[bib0020] Rapelli G., Giusti E.M., Donato S., Parise M., Pagani A.F., Pietrabissa G., Bertoni A., Castelnuovo G. (2023). « The heart in a bag » : the lived experience of patient-caregiver dyads with left ventricular assist device during cardiac rehabilitation. Front. Psychol..

[bib0021] Regamey D.J., Kirsch P.M., Tozzi P., Barras D.N., Marcucci C., Liaudet P.L., Hullin R. (2018). Options thérapeutiques dans l’insuffisance cardiaque avancée: place de l’assistance ventriculaire gauche permanente (LVAD). Rev. Médicale Suis..

[bib0022] Saeed D., Feldman D., Banayosy A.E., Birks E., Blume E., Cowger J., Hayward C., Jorde U., Kremer J., MacGowan G., Maltais S., Maybaum S., Mehra M., Shah K.B., Mohacsi P., Schweiger M., Schroeder S.E., Shah P., Slepian M., D’Alessandro D. (2023). The 2023 international society for heart and lung transplantation guidelines for mechanical circulatory support: a 10- year update. J. Heart. Lung. Trans. Plant.

[bib0023] Stahl E.P., Dickert N.W., Cole R.T., Laskar S.R., Morris A.A., Smith A.L., Vega J.D., Gupta D. (2019). Decisional regret in left ventricular assist device patient-caregiver dyads. Heart Lung.

[bib0024] Standing H.C., Exley C., MacGowan G.A., Rapley T. (2018). ’We’re like a gang, we stick together’ : experiences of ventricular assist device communities. Eur. J. Cardiovasc. Nurs..

[bib0025] Streur M.M., Auld J.P., Liberato A.C.S., Beckman J.A., Mahr C., Thompson E.A., Dougherty C.M. (2020). Left ventricular assist device caregiver experiences and health outcomes : a systematic review of qualitative and quantitative studies. J. Card. Fail..

[bib0026] Talbot N. (2017). Fortin, M- F. et Gagnon, J. (2016). Fondements et étapes du processus de recherche : méthodes quantitatives et qualitatives (3e édition). Montréal, Québec : chenelière éducation. Rev. Des. Sci. De L’éducat..

[bib0027] Wasilewski G., Wiśniowska-Śmiałek S., Górkiewicz-Kot I., Milaniak I., Kaleta M., Hymczak H., Tomsia P., Wierzbicki K. (2024). Transplantation Proceedings.

[bib0028] Yin R.K. (2018).

