# Supplementary material for: Exploring patient, caregiver and care team experiences of destination therapy Left Ventricular Assist Device (LVAD): A multiple case qualitative study protocol
Source: Int J Nurs Stud Adv. 2026 May 23;11:100572. doi: 10.1016/j.ijnsa.2026.100572 (PMC13241731; doi:10.1016/j.ijnsa.2026.100572)
Supplement: Supplementary file 1 [file mmc1.pdf]

COMMISSION CANTONALE
D'ÉTHIQUE DE LA RECHERCHE
SUR L'ÊTRE HUMAIN
CER-VD
Av. de Chailly 23
1012 Lausanne

Pre Claudia Ortoleva Bucher
Haute école de santé Fribourg
HES-SO - Heds FR
Rte des Arsenaux 16A
1700 Fribourg

Lausanne, le 24.04.2026
Réf. SG/ccg/ac

**Décision de la Commission cantonale (VD) d'éthique de la recherche sur l'être humain (CER-VD)**

| | |
|---|---|
| **Project-ID** | 2026-00386 |
| **Titre du projet** | Vécu, besoins et stratégies d'adaptation de la dyade personne porteuse d'un LVAD-DT (left ventricular assist device – destination therapy) et proche aidant·e : une étude de cas multiples |
| **Travail de master/de thèse de** | Tania Dominguez Canosa |
| **Direction du projet** | Pre Claudia Ortoleva Bucher |
| **Promoteur** | Heds Fribourg, Pre Claudia Ortoleva Bucher |
| **Centres** | Pre Claudia Ortoleva Bucher, Heds Fribourg, Fribourg |

**Décision**

☒ Autorisation accordée
Cette autorisation est valable pour la durée annoncée de l'étude mais au maximum pour 5 ans à compter de la date de la présente décision.

☐ Autorisation avec charges
Cette autorisation est valable pour la durée annoncée de l'étude mais au maximum pour 5 ans à compter de la date de la présente décision.

☐ En l'état, l'autorisation ne peut pas être accordée

☐ Autorisation non accordée

☐ Non entrée en matière

**Remarques**

**Formulaire d'information et de consentement**

– Ajouter la durée de conservation des données collectées et des enregistrements dans l'ICF pour les professionnels (point 9, lettre f, de la décision initiale de la CER-VD).

– Préciser qui aura accès à ces enregistrements, en particulier pour les professionnels de la santé (point 9, lettre g, de la décision initiale de la CER-VD).

– Dans votre réponse au point 3, lettre b, i, vous avez indiqué que "Les participant·e·s seront également informé·e·s qu'ils·elles peuvent choisir de ne pas aborder certains sujets en présence de l'autre personne". Veuillez ajouter cette information dans les formulaires d'information des patients et proches.

– Supprimer la mention des échantillons de l'ensemble des ICFs, car elle figure encore dans les titres des sections 8 et 8.2 (Point 9, lettre d, de la décision initiale de la CER-VD), et ajouter le N° BASEC (2026-00386) sur les feuilles de consentement.

**Classification**

☒ Projet de recherche au sens de l'ORH — Catégorie : A

- ☒ recherche sur des personnes
- ☐ réutilisation du matériel biologique ou des données personnelles liées à la santé
- ☐ sur des personnes décédées
- ☐ sur des embryons et des fœtus
- ☐ avec rayonnements ionisants

**Procédure de décision**

☐ Procédure ordinaire ☐ Procédure simplifiée ☒ Procédure présidentielle

La Commission certifie se conformer aux principes ICH GCP.

**Taxes et émoluments**

Déjà facturé.

**Voies de recours**

La présente décision peut faire l'objet d'un recours au Tribunal cantonal, Cour de droit administratif et public. L'acte de recours doit être déposé auprès du Tribunal cantonal dans les **30 jours** suivant la communication de la décision attaquée ; il doit être signé et indiquer les conclusions et motifs du recours. La décision attaquée est jointe au recours. Le cas échéant, ce dernier est accompagné de la procuration du mandataire.

**Copie pour information à :**

☐ OFSP

☒ Autre(s) Rebetez Nathalie, nathalie.rebetez@hefr.ch

**Signature**

p.o. Arthur Zinn, secrétaire général

Susanna Gerber, PhD
Vice-présidente

**Annexes :** -Obligations du requérant
-Signification des décisions possibles
-Liste des documents soumis les 18.02.2026, 07.04.2026 et 22.04.2026

## Annexes

**Obligations du requérant (promoteur ou direction du projet) :**

**Soumission de documents** : les documents modifiés et les nouveaux documents relatifs à l'étude/au projet de recherche sont soumis via le dossier existant. Les documents qui ne sont plus valides sont effacés et remplacés par les nouveaux. Les documents révisés doivent être soumis une fois en mode « suivi des modifications » et une fois en mode « modifications acceptées » (« track changes » et « clean »). Les documents d'information et de consentement ainsi que le protocole doivent être transmis dans un format permettant la recherche (PDF navigable) ou scannés avec une fonction OCR (Optical Character Recognition). Le cas échéant, les documents révisés sont également mis à disposition des autorités compétentes pour approbation.

Remarque : La commission d'éthique compétente examine, dans le cadre du processus d'autorisation, les feuilles d'information et déclarations de consentement dans une des langues officielles suisses: allemand, français ou italien. La commission d'éthique ne fait qu'accuser réception des feuilles d'information et déclarations de consentement écrites dans d'autres langues. Le promoteur ou la direction du projet est responsable de la traduction correcte des documents.

**Obligations d'annonce** : Les obligations d'annonce (p.ex. d'événements indésirables, d'interruption d'étude) et de soumission pour autorisation des modifications essentielles obligatoires s'appliquent (Ordonnances). Le rapport final est à remettre à la commission d'éthique compétente dans un délai d'une année à compter de la fin ou de l'arrêt de l'étude.

Pour les essais cliniques de cat. B et C, la demande doit être présentée à la deuxième autorité compétente en matière d'autorisation (à Swissmedic, respectivement à la commission d'éthique) dans les deux ans suivant l'octroi de l'autorisation par la première autorité compétente en matière d'autorisation (art. 23 al.1bis OClin).

**Devoir d'enregistrement** : Le promoteur d'un essai clinique doit procéder à l'enregistrement dans un registre primaire reconnu par l'OMS ou dans le registre de la bibliothèque médicale nationale des Etats-Unis d'Amérique (clinicaltrials.gov) puis indiquer le numéro de l'étude sur le portail BASEC. Le transfert des données vers le Swiss National Clinical Trials Portal (HumRes) est effectué automatiquement suite à l'autorisation de l'étude par la commission d'éthique. Les données relatives à l'essai clinique figurant sur les deux registres sont accessibles au public. Swissethics publie également sur son site des informations sur chaque étude ayant reçu une autorisation.

**Signification des décisions possibles**

**Autorisation accordée** : L'étude peut commencer selon le plan de recherche accepté. Elle doit être menée dans le cadre des dispositions légales en vigueur. D'autres obligations d'autorisation (Swissmedic/OFSP) doivent être respectées.

**Autorisation avec charges** : L'étude peut commencer selon le plan de recherche accepté. Elle doit être menée dans le cadre des dispositions légales en vigueur. Les charges doivent être remplies dans un délai de 30 jours. Les documents modifiés seront réévalués en procédure présidentielle. D'autres obligations d'autorisation (Swissmedic / OFSP) doivent être respectées.

**En l'état, l'autorisation ne peut pas être accordée** : L'étude ne peut pas commencer. Prière de répondre point par point aux conditions de la commission d'éthique et de lui faire parvenir les documents révisés avec les modifications apparentes et la mention de la date de la nouvelle version.

**Autorisation non accordée** : L'étude ne peut pas commencer dans sa forme actuelle. Une nouvelle soumission reste possible.

**Non entrée en matière** : Justification, voir ci-dessus, par exemple la commission d'éthique n'est pas juridiquement compétente pour accorder une autorisation ou l'étude ne nécessite pas d'autorisation.

**Liste des documents soumis**

| Pre Claudia Ortoleva Bucher, Heds Fribourg, Fribourg | | |
|---|---|---|
| nom du fichier | date du fichier | version |
| **1. Cover Letter** | | |
| ex-needs-lvad-dt-coverletter.pdf | 12/02/2026 | |
| **2. Synopsis of the study plan** | | |
| see doc/cat: **4**, page/ref: **1-2** | | |
| **3. Participant information sheet and informed consent (ICF)** | | |
| ex-needs-lvad-dt-info-cons-patients-trackchange-v2.docx | 02/04/2026 | 2 |
| ex-needs-lvad-dt-info-cons-proches-trackchange-v2.docx | 02/04/2026 | 2 |
| ex-needs-lvad-dt-info-cons-profess-clean-v2.docx | 02/04/2026 | 2 |
| ex-needs-lvad-dt-info-cons-proches-clean-v2.docx | 02/04/2026 | 2 |
| ex-needs-lvad-dt-info-cons-patients-clean-v2.docx | 02/04/2026 | 2 |
| ex-needs-lvad-dt-info-cons-profess-trackchange-v2.docx | 02/04/2026 | 2 |
| **4. Study plan (protocol), signed and dated** | | |
| ex-needs-lvad-dt-procotolecer-clean-v2.pdf | 02/04/2026 | 2 |
| ex-needs-lvad-dt-procotolecer-trackchange-v2.pdf | 02/04/2026 | 2 |
| **6. Investigator's CV, dated** | | |
| cvco-eng-2026.pdf | 10/02/2026 | |
| cv-lp-maya-zumstein-shaha-3-13-2026.pdf | 02/04/2026 | |
| cv-taniadominguez.pdf | 02/04/2026 | |
| rebetez-n-cv.pdf | 12/02/2026 | |
| angeloz-d-cv.pdf | 02/04/2026 | |
| **11. Other documents handed over to study participants** | | |
| ex-needs-lvad-dt-accordtransmissionscoordonnees-v1.pdf | 12/02/2026 | 1 |
| **12. Details on nature and scope/value of compensation for participants** | | |
| There is no compensation for the participation in this study | | |
| **14. Information on secure handling of biological material and personal data, and in particular on the storage thereof** | | |
| see doc/cat: **4**, page/ref: **lignes 419-487** | | |
| **39. Miscellaneous / Varia** | | |
| ex-needs-lvad-dt-guides-entretien-trackchanges-v2.docx | 02/04/2026 | 2 |
| ex-needs-lvad-dt-questionnairesociodem-patients-trackchange-v1.docx | 02/04/2026 | 1 |
| ex-needs-lvad-dt-organigramme.pdf | 02/04/2026 | 1 |
| 2026-00386-form-rep-20260402.docx | 02/04/2026 | 1 |
| ex-needs-lvad-dt-lettre-de-soutien-chuv.pdf | 23/03/2026 | 1 |
| ex-needs-lvad-dt-questionnairesociodem-professionnels-v1.docx | 02/04/2026 | 1 |
| ex-needs-lvad-dt-guides-entretien-clean-v2.docx | 02/04/2026 | 2 |
| ex-needs-lvad-dt-questionnairesociodem-pa-v1.docx | 02/04/2026 | 1 |
| ex-needs-lvad-dt-questionnairesociodem-patients-clean-v1.docx | 02/04/2026 | 1 |
